# Supplementary material for: Intrauterine Blood Plasma Platelet-Therapy Mitigates Persistent Breeding-Induced Endometritis, Reduces Uterine Infections, and Improves Embryo Recovery in Mares
Source: Antibiotics (Basel). 2021 Apr 23;10(5):490. doi: 10.3390/antibiotics10050490 (PMC8146422; doi:10.3390/antibiotics10050490)
Supplement: Supplementary file 1 [file antibiotics-10-00490-s001.zip › antibiotics-1175412-supplementary.pdf]

Supplement material

Intrauterine Blood Plasma Platelet-Therapy Mitigates Persistent Breeding-Induced Endometritis, Reduces Uterine Infections, and Improves Embryo Recovery in Mares

Lorenzo G. T. M. Segabinazzi [1,2,5], Igor F. Canisso [1,*], Giorgia Podico [1], Lais L. Cunha [1], Guilherme Novello [1,2], Michael F. Rosser [3], Shavahn C. Loux [4], Fabio S. Lima [1,6] and Marco A. Alvarenga [2]

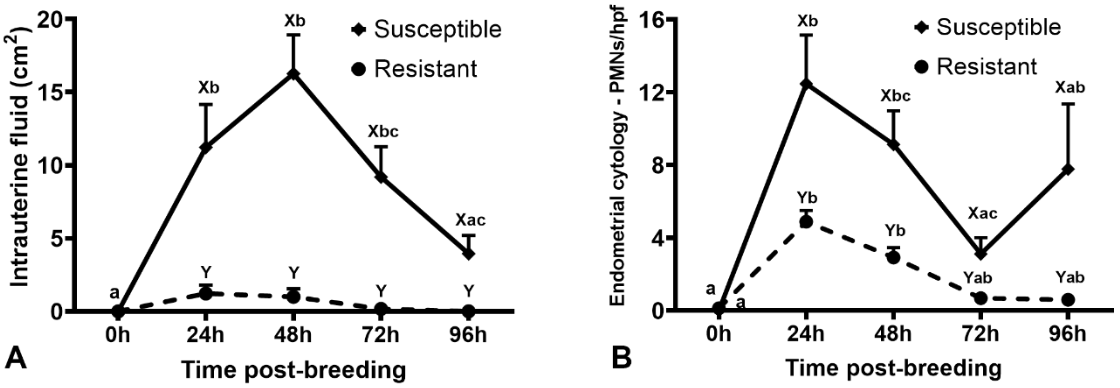

Figure S1. The post-breeding inflammatory response in mares after the sperm challenging. Mares were categorized as susceptible (n = 12) and resistant (n = 7) to persistent-breeding induced endometritis (PBIE) immediately pre-(0h) and post-sperm challenging (24–96h). (A) Intrauterine fluid accumulation measured at the uterine bifurcation; (B) Mean PMNs counted in endometrial cytology in five high-power fields (hpf). Different superscripts denote the effects of time (^{abc}) and differences between mares within each time point (^{XY}) ($P < 0.05$).

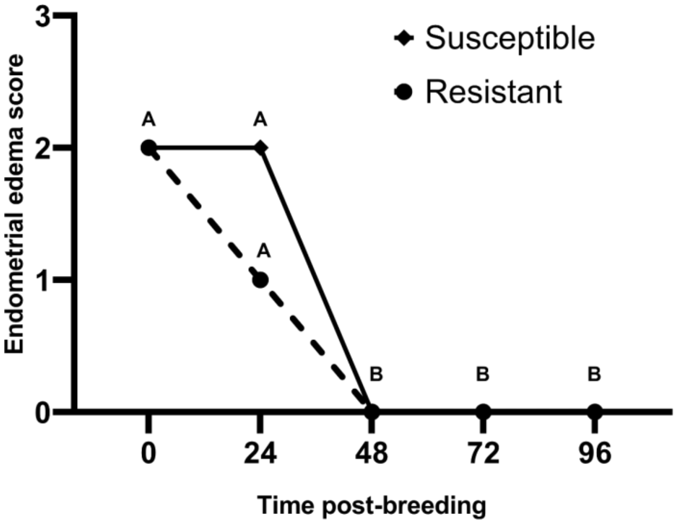

Figure S2. Endometrial edema score (median) in mares after the sperm challenging. Mares were categorized as susceptible (n = 12) and resistant (n = 7) to persistent-breeding induced endometritis (PBIE) immediately pre-(0h) and post-sperm challenging (24–96h). Score 0, No edema; 1, Mild edema; 2, Moderate edema; 3, Evident edema; 4, Exacerbated edema. Different superscripts denote the effects of time (^{A,B}) ($P < 0.05$).

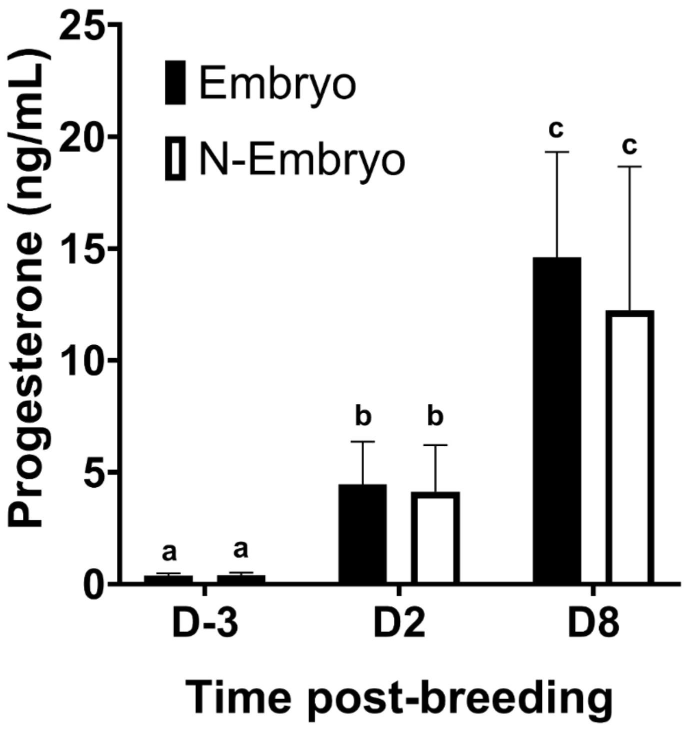

Figure S3. Circulating progesterone concentrations pre- and post-ovulation in mares susceptible to persistent breeding-induced endometritis with a positive (Embryo) or negative (N-Embryo) embryo flushing at eight days post-ovulation. Assessments were carried out from the early onset of estrus (D-3), 48 h post-ovulation (D2) and on the day of embryo flushing, eight days post-ovulation (D8).

Table S1. Semen parameters for the breeding doses used for breeding mares assigned to (Control), platelet-rich (PRP), or -poor plasma (PPP). All ejaculates (n = 26) were obtained from a single fertile stallion.

Parameters	Control	PRP	PPP
Volume (mL)	13.3 ± 1.0	15.4 ± 1.3	14.7 ± 0.9
Total sperm concentration (× 10^9)	2.7 ± 0.14	2.5 ± 0.15	2.6 ± 0.9
Total motility (%)	80.5 ± 1.7	80.3 ± 2.1	81.0 ± 1.0
Progressive motility (%)	74.0 ± 2.0	74.4 ± 2.0	75.0 ± 1.4

Data represented as Mean ± SEM.
